# Use of a Smartphone to Gather Parkinson's Disease Neurological Vital Signs during the COVID-19 Pandemic

**DOI:** 10.1155/2021/5534282

**Published:** 2021-04-10

**Authors:** Jay L. Alberts, Mandy Miller Koop, Marisa P. McGinley, Amanda L. Penko, Hubert H. Fernandez, Steven Shook, Robert A. Bermel, André Machado, Anson B. Rosenfeldt

**Affiliations:** ^1^Cleveland Clinic, Lerner Research Institute, Department of Biomedical Engineering, Cleveland, OH, USA; ^2^Cleveland Clinic, Neurological Institute, Center for Neurological Restoration, Cleveland, OH, USA; ^3^Cleveland Clinic, Neurological Institute, Mellen Center for Multiple Sclerosis, Cleveland, OH, USA; ^4^Cleveland Clinic, Lerner College of Medicine, Cleveland, OH, USA

## Abstract

**Introduction:**

To overcome travel restrictions during the COVID-19 pandemic, consumer-based technology was rapidly deployed to the smartphones of individuals with Parkinson's disease (PD) participating in a 12-month exercise trial. The aim of the project was to determine the feasibility of utilizing a combined synchronous and asynchronous self-administered smartphone application to characterize PD symptoms.

**Methods:**

A synchronous video virtual visit was completed for the administration of virtual Movement Disorder Society-Unified Parkinson's Disease Rating Scale III (vMDS-UPDRS III). Participants asynchronously completed a mobile application consisting of a measure of upper extremity bradykinesia (Finger Tapping Test) and information processing.

**Results:**

Twenty-three individuals completed the assessments. The mean vMDS-UPDRS III was 23.65 ± 8.56 points. On average, the number of taps was significantly greater for the less affected limb, 97.96 ± 17.77 taps, compared to the more affected, 89.33 ± 18.66 taps (*p* = 0.025) with a significantly greater number of freezing episodes for the more affected limb (*p* < 0.05). Correlation analyses indicated the number of errors and the number of freezing episodes were significantly related to clinical ratings of vMDS-UPDRS III bradykinesia (Rho = 0.44, *p* < 0.01; *R* = 0.43, *p* < 0.01, resp.) and finger tapping performance (Rho = 0.31, *p* = 0.03; Rho = 0.32, *p* = 0.03, resp.). *Discussion*. The objective characterization of bradykinesia, akinesia, and nonmotor function and their relationship with clinical disease metrics indicate smartphone technology provides a remote method of characterizing important aspects of PD performance. While theoretical and position papers have been published on the potential of telemedicine to aid in the management of PD, this report translates the theory into a viable reality.

## 1. Introduction

The coronavirus infectious disease (COVID-19) results from infection from the novel SARS-CoV-2 and causes mild-moderate respiratory infection in most individuals; however, the infection can cause severe infection and death in some people. COVID-19 was first identified in late 2019, and with global concern escalating, the impact on the United States began in March 2020. This resulted in sweeping travel restrictions and limited in-person gathering in an attempt to decrease transmission rates. The emergence of COVID-19 catapulted telemedicine as an essential service for patients across all domains of medicine to aid in disease management and contain virus spread. The World Health Organization defines telemedicine as “the delivery of health care services, where distance is a critical factor, by all health care professionals using information and communication technologies *for the exchange of valid information* for diagnosis, treatment, and prevention of disease and injuries, research and evaluation” [1]. For decades, telemedicine has promised to aid in the delivery of care and monitoring of research outcomes for neurological patients [2] with greater convenience and comfort [3]; however, direct experience and prospective data utilizing synchronous and asynchronous virtual visits are lacking. There have been several reports of remote smartphone monitoring in Parkinson's disease (PD); however, several of these reports have neglected assessing nonmotor function [4, 5] while others are time prohibitive by taking 20 minutes to administer [6]. Previously, we utilized the inertial measurement unit (IMU) data derived from smartphones to characterize postural stability and gait [7–13] and executive functioning [14–16], in neurological patients as part of routine in-person care with improved efficiencies in time and cost [17–20]. COVID-19 catalyzed our translation of this validated mobile technology from the clinic to the patient's home.

COVID-19 travel restrictions prevented in-person data collection sessions in a longitudinal PD exercise trial. Rather than sacrificing final assessment data, a remote model of data collection was implemented where consumer-based technology was rapidly deployed to a group of individuals with PD participating in a 12-month pragmatic exercise trial. The goal of the remote assessments was to complete the 12-month assessments of motor and nonmotor function within the confines of a clinical study. We sought to determine the feasibility of gathering data from a synchronous virtual visit and asynchronous self-administered smartphone application and evaluate the relationship between smartphone outcomes and clinical assessments.

## 2. Methods

Participants across five community-based exercise sites in northern Washington and central Colorado were enrolled in an observational trial evaluating the long-term effects of exercise on PD progression in individuals enrolled in a year-round, disease-specific, community-based cycling class, Pedaling for Parkinson's. In-person collection of motor and nonmotor performance was planned on three occasions: baseline, 6 months, and 12 months while off antiparkinsonian medication. The assessments consisted of a variety of upper and lower extremity motor tasks, as well as nonmotor assessments. The initiation of trial activities began prior to the COVID-19 pandemic, and therefore all baseline assessments were completed, in-person, as originally planned.

In Washington, data at all three time points (baseline, 6 months, and 12 months) were collected in-person by January 2020, prior to the beginning of the COVID-19 pandemic in the United States. In the spring of 2020, COVID-19 travel restrictions prevented in-person data collection for 12-month assessments in Colorado. In lieu of an in-person visit, a two-part remote assessment was completed: (1) a synchronous clinical video visit with a physical therapist (PT) and (2) an asynchronous data collection session with the Cleveland Clinic Digital Neurological Vital Signs (DNVS) smartphone application. The Cleveland Clinic IRB approved remote data collection in response to COVID-19.

### 2.1. Participants

Thirty-eight individuals with PD were successfully contacted at the Colorado sites; 23 had a suitable iPhone and installed and completed DNVS application and were included in the final analysis. Fifteen participants were unable to download the DNVS application. The primary reasons for being unable to download the DNVS app were no access to an Apple iPhone (*n* = 9), poor understanding of downloading applications, or lacking motivation to download (*n* = 5), and one patient was able to download, but cognitive issues prevented completion of DNVS modules (*n* = 1). Consistent with the original protocol, participants withheld antiparkinsonian medication 12 hours prior to the assessments. Participant demographics at the time of the 12-month assessment are displayed in Table 1.

### 2.2. Outcome Measures

#### 2.2.1. Design of the DNVS Application

The DNVS application was developed using the native iPhone operating system (iOS) in the programming language of Swift. It consisted of two modules: (1) Finger Tapping Test (FTT) to assess upper extremity bradykinesia and (2) Processing Speed Test (PST) to assess cognition, specifically, processing speed. Both modules are described in further detail below. Data were sampled at a rate of 60 Hz and outcome metrics were automatically processed on the device. Data were encrypted on the device to protect subject identity, and no protected health information was stored in the module application. The deidentified data were transmitted to health information privacy (HIPPA) secure cloud managed by the Cleveland Clinic's Information Technology Department. Members of the study team then downloaded data for subsequent analysis.

#### 2.2.2. Synchronous and Asynchronous Assessments

The project employed a series of synchronous and asynchronous visits. Synchronous assessments referred to the subject performing the task at the same time the rater was grading/assessing the task (i.e., virtual Movement Disorder Society–Unified Parkinson's disease Rating Scale III (vMDS-UPDRS) and mobility assessment). Asynchronous assessments referred to the subject performing the task independently on their mobile device (i.e., FTT and PST). The asynchronous DNVS data were processed in the manner described above.

#### 2.2.3. vMDS-UPDRS III (Synchronous)

Via a video platform, the PT with six years of experience administering the MDS-UPDRS III performed the vUPDRS III, sans rigidity and postural instability items [21–23]. Symptoms were subdivided into: upper extremity (UE) score (items: 3.4–3.6, 3.15–3.17a-b), lower extremity score (items: 3.7-3.8, 3.17c-d), UE bradykinesia (items 3.4–3.6), UE tremor (items 3.15–3.17a-b), finger tapping (item 3.4), and postural instability and gait dysfunction (PIGD) subscore included items (3.9–3.13).

### 2.3. Functional Mobility (Synchronous)

Functional mobility was assessed via the Timed up and go (TUG) [24], where the individual stands from a chair, walks 10 feet, turns, returns to the chair, and sits. Patients measured the appropriate distance in their home and completed the task while being timed via a stopwatch by the PT via a video platform.

### 2.4. Smartphone Upper Extremity Bradykinesia (Asynchronous)

**Figure 1:**
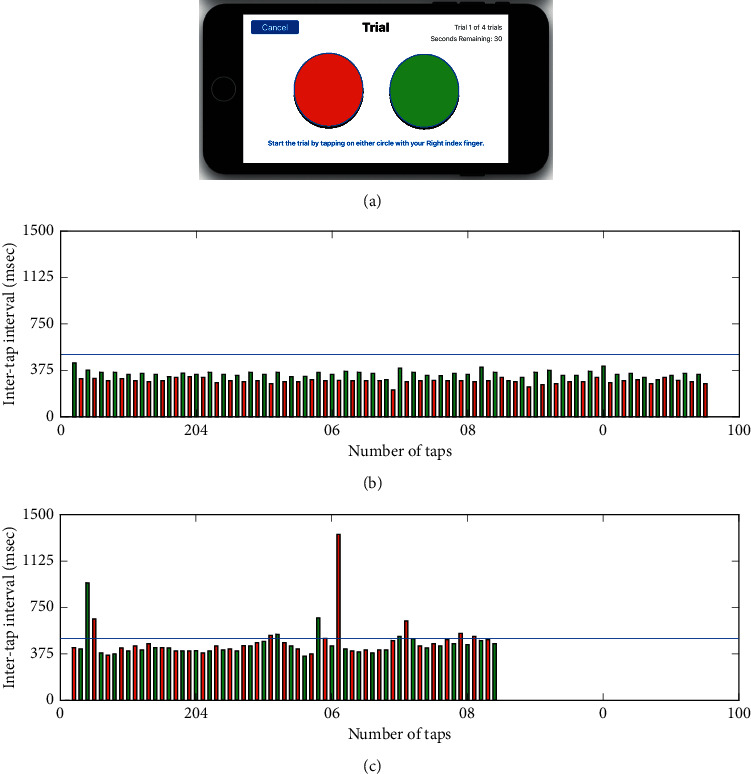
(a) Screenshot of the display from the FTT application on an iPhone. Representative data from one participant performing FTT test with their less affected (b) and more affected (c) hands. Each bar represents the time duration (ms) between the onset of consecutive taps (intertap interval) with the left target shown in red and the right one in green. Errors were defined as consecutive taps on the same target. Intertap intervals greater than 500 msec were classified as a freeze (blue line denotes threshold for a freeze). The more affected side performed a lower number of total taps compared to the less affected hand (95 vs. 64 taps, resp.), with a longer average intertap interval (461.6 vs. 315.1 ms, resp.), committed an increased number of errors (1 vs. 0 errors, respectively), and exhibited a greater number of freezing episodes (11 vs. 0, resp.).

Upper extremity function was evaluated using a reciprocal target-directed FTT (Figure 1) [25]. Following uniform instructions provided within the DNVS application, participants used their index finger to continuously and quickly tap between targets for 30 seconds; two trials per limb. The number of taps, intertap interval (time interval between the onset of a tap and the onset of the next consecutive tap), and errors (double tapping the same target) were automatically calculated by the DNVS. The average of two trials per limb was used in the analyses.

#### 2.4.1. Nonmotor Performance (Asynchronous)

Our previously validated PST, a symbol-digit matching test, was used to quantify information processing, attention, and working memory [15, 16]. In this module, individuals are asked to match numbers with their corresponding symbols while referring to a matching key.

### 2.5. Statistical Analysis

A one-sample Kolmogorov–Smirnov test was performed on each of the outcome metrics to test for normal distribution. Paired *t*-tests or Wilxcon signed-rank tests, as appropriate, were utilized to evaluate differences across outcomes as a function of the self-reported more and less affected side. Pearson or Spearman rank correlation analyses, as appropriate, were performed to determine the level of agreement between DNVS outcomes (FTT, TUG, and PST) and vMDS-UPDRS III. Statistical analysis was conducted using R (version 4.0.2) with an alpha of 0.05.

## 3. Results

All 23 participants were able to complete assessment modules except for two individuals who were unable to complete the PST secondary to cognitive issues.

### 3.1. vUPDRS III

The mean vUPDRS III was 23.65 ± 8.56. Clinical ratings for the more and less affected side for clinical subscores are provided in Table 2. The more affected side was significantly worse for all clinical ratings compared to the less affected side (*p* < 0.05), except for tremor.

### 3.2. Smartphone Bradykinesia Is Related to vMDS-UPDRS III and Laterality

**Figure 2:**
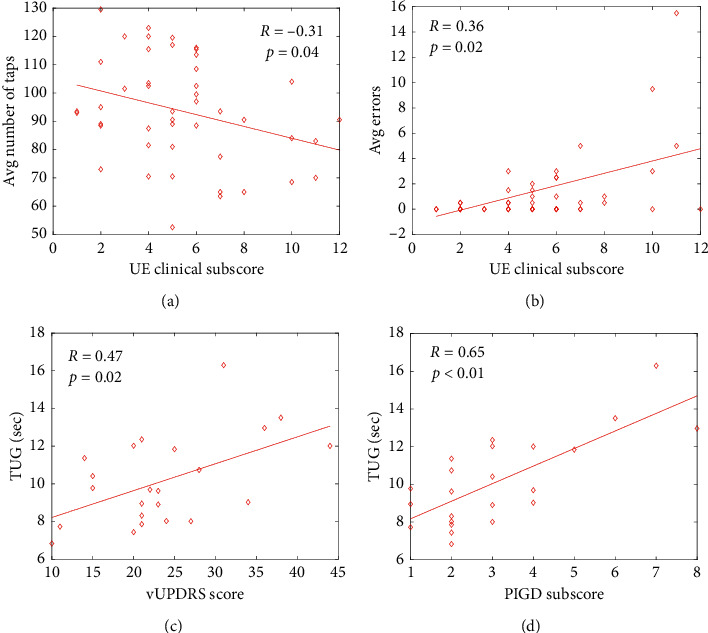
Finger Tapping Test measures: (a) the number of taps and (b) the number of errors were significantly related to upper extremity (UE) PD severity score measured by the vMDS-UPDRS III. Timed up and go trial times (c, d) were significantly related to total vMDS-UPDRS-III score, and clinical measures of postural and gait deficits (PIGD) measured by the vMDS-UPDRS III, Spearman rank correlation analyses, *p* < 0.05 for a–d.

The number of taps was significantly greater for the less affected limb 97.96 ± 17.77 taps compared to the more affected, 89.33 ± 18.66 taps (*p*=0.025; Figure 1; Table 2). The time interval between taps was used to estimate the freezing or pauses in movement (intervals 500+ ms) [26]. The number of freezing episodes was significantly greater for the more affected compared to the less affected limb (*p* < 0.05, Table 2). Lateralized clinical and quantitative data of the more and less affected limbs were paired and a Spearman rank correlation analysis revealed that participants with greater upper extremity disease severity on the vMDS-UPDRS III completed fewer taps during the FTT (Rho = −0.31, *p* = 0.04) and committed more errors (double taps) (Rho = 0.36, *p*=0.02). Correlation analyses indicated the number of errors committed and the number of freezing episodes were significantly related to clinical ratings of vMDS-UPDRS III bradykinesia (Rho = 0.44, *p* < 0.01; *R* = 0.43, *p* < 0.01, resp.) and finger tapping performance (Rho = 0.31, *p*=0.03; Rho = 0.32, *p*=0.03) (Figure 2).

### 3.3. Functional Mobility Is Related to vMDS-UPDRS III

The average time to complete the TUG was 10.1 ± 2.3 seconds. TUG time was significantly correlated to overall disease severity, total vMDS-UPDRS III (Rho = 0.47, *p*=0.02), lower extremity function on the vMDS-UPDRS III (Rho = 0.42, *p*=0.05), and posture and gait impairments on the vMDS-UPDRS III (PIGD; Rho = 0.65, *p* < 0.01).

### 3.4. Cognitive Function

The average number of correct responses on the PST was 36.05 ± 8.87, while incorrect responses were 0.95 ± 1.28. There was no relationship between the number of correct responses and any vMDS-UPDRS or FTT outcomes.

## 4. Discussion

The physical examination is the cornerstone for evaluating and treating PD. The objective characterization of bradykinesia, akinesia, and functional mobility using technology such as the DNVS application may be critical for the broad adoption of telemedicine in clinical and research settings. We quickly and securely deployed smartphone technology to a group of older adults with PD to preserve the integrity of a 12-month clinical trial. The data from the DNVS application provided objective and quantitative data related to the cardinal symptoms of PD that exhibited agreement with traditional clinical ratings.

Freezing of movement is a debilitating aspect of PD, yet it can be difficult to elicit during in-person clinical visits [27] and certainly challenging as part of a virtual assessment. Furthermore, clinical rating of freezing varies widely between clinicians and does not always correlate with objective metrics [28]. The FTT was successful in eliciting movement freezing and precisely and automatically quantifying its occurrence. Hence, the use of a relatively simple motor task combined with the data recording capabilities of a mobile device provides unique insight into PD motor function. Demonstrating agreement between clinical and derived biomechanical outcomes combined with the success in self-administration provides rationale for the utilization of applications that leverage consumer devices in both a research and clinical environment. The use of consumer electronic technology coupled with self-administered assessments has the potential to facilitate the use of telemedicine outside of a pandemic by collecting objective, serial data that can potentially aid with clinical decision-making. For example, the DNVS application could be taken throughout the day to quantify fluctuations in motor and nonmotor function and response to medication, specifically regarding “off” periods when antiparkinsonian medication becomes less effective.

The concept of telemedicine was introduced in the early 1960s and has largely failed to gain acceptance due to lack of reimbursement, cost of hardware and software necessary to connect the patient to provider, and insufficient bandwidth (for perspectives over the decades, see Jerant 1998 [29], Grigsby 1998 [2], and Dorsey 2018 [30]). The utilization of smartphone applications addresses these obstacles that slowed the implementation of telemedicine. The leveraging of pervasive, consumer-based technology essentially eliminates the cost of technology as a barrier for patients and hospital systems. Self-administration of the application by the patient in their home addresses the recent call that telemedicine be convenient and comfortable for PD patients [3]. Importantly, we ensured confidentiality through application development by employing information technology personnel with previous experience programming mobile devices and cloud computing for HIPPA compliance. The collaborative nature of these interactions facilitated the use of DVNS in a research project that otherwise would have been compromised as a result of COVID-19. Consumer-available technology that is user-friendly toward older individuals with neurological disease has the potential to evolve telemedicine beyond a video chat by objectively tracking motor and nonmotor data. By augmenting the traditional subjective video visit, telemedicine can be expanded to potentially more precisely improve PD-medication titration and deep brain stimulation programming.

Efforts are currently underway to create an Android version of DNVS to increase the availability of the technology to patients who may not have an iOS device. This is a critical step toward widespread clinical use and integration. We are currently working with other neurological centers to deploy the DNVS app to other neurological populations, including individuals with dementia, Multiple Sclerosis (MS), and community-dwelling adults who experience falls, as much of the clinical data collected in the DNVS application (functional mobility, upper extremity function, and information processing) are important in managing other patient populations. Our goal is to establish a standardized core group of physical and cognitive telemedicine assessments that could be used to aid in the management of multiple neurological patients. Telemedicine is a viable option for many individuals, as 89 percent of individuals in the United States own a smartphone or other Internet access device [31], and the COVID-19 pandemic has demonstrated that more individuals are equipped to perform virtual visits than was initially anticipated. Moving beyond subjective video visits in the treatment of neurological disease is a critical step in propelling telemedicine to a preferred method of medical visits.

## Figures and Tables

**Table 1:** Participant demographics (*N* = 23).

Characteristic	Value
Age, years	68.4 (7.8)
Race, white	22 (95.7%)
Sex, male	15 (65.2%)
Education, years	18.4 (2.1)
Upper extremity more affected by PD	
Right	8 (34.8%)
Left	15 (65.2%)
Disease duration, years	6.1 ± 4.5
Hoehn and Yahr	
II	19 (82.6%)
III	4 (17.4%)
Levodopa Equivalent Daily Dose (mg)	699.8 ± 368.3

Data presented as mean ± SD or *n* (%).

**Table 2:** Summary statistics for virtual MDS-UPDRS III ratings and performance on the digital neurological vital signs.

		Total		More affected		Less affected	
		Mean	SD	Mean	SD	Mean	SD
vMDS-UPDRS-III (pts)	UE subscore	10.78	4.1	**6.39**	**2.61**	**4.39**	**2.5**
	LE subscore	4.48	2.5	**2.78**	**1.65**	**1.7**	**1.29**
	Bradykinesia subscore-UE	8.52	3.01	**4.87**	**1.74**	**3.65**	**1.61**
	Tremor subscore-UE	2.26	2.51	1.57	1.8	0.7	1.4
	Finger tapping score	3.43	1.24	**1.91**	**0.73**	**1.52**	**0.67**
	mUPDRS-III	23.65	8.56				
	PIGD subscore	3.13	1.87				

FTT	Number of taps	93.64	18.22	**89.33**	**18.66**	**97.96**	**17.77**
	Number of errors	1.57	3.19	1.26	2.36	1.87	3.85
	Intertap interval (msec)	326.72	70.35	**344.69**	**81.49**	**308.74**	**57.08**
	Number of freezes	3.22	7.7	**4.63**	**10.64**	**1.8**	**2.33**

TUG	Total trial time (sec)	10.16	2.34				

PST	Total correct	36.05	8.87				
	Total incorrect	0.95	1.28				

^*∗*^ *p* < 0.05; bold values indicate a significant difference between more and less affected sides. FTT, Finger Tapping Test; LE, lower extremity, PST, Processing Speed Test; UE, upper extremity; TUG, timed up and go test; and virtual Movement Disorder Society-Unified Parkinson's Disease Rating Scale III (vMDS-UPDRS III).

## Data Availability

The data are stored in a secured database at the Cleveland Clinic and can be made available upon request.
